# Rhythms of life: melatonin, nutrition, sleep, and antioxidant strategies for healthy aging

**DOI:** 10.3389/fnins.2026.1736978

**Published:** 2026-03-04

**Authors:** Cristina Manuela Drăgoi, Ion-Bogdan Dumitrescu, Alina Crenguţa Nicolae

**Affiliations:** 1 Department of Biochemistry, Faculty of Pharmacy, Carol Davila University of Medicine and Pharmacy, Bucharest, Romania; 2 Department of Physics and Informatics, Faculty of Pharmacy, Carol Davila University of Medicine and Pharmacy, Bucharest, Romania

**Keywords:** aging, chronobiology, chrononutrition, circadian rhythm, dietary and lifestyle interventions, melatonin, molecular clocks, sleep

## Abstract

Circadian rhythms orchestrate nearly all physiological and behavioral functions, and their deterioration with aging contributes to sleep disruption, cognitive decline, and increased vulnerability to disease. Melatonin, the primary hormonal signal of darkness, plays a central role in maintaining circadian synchrony, regulating sleep, and exerting antioxidant and neuroprotective effects. This review provides an integrative synthesis of current evidence on the multi-layered role of melatonin in aging, encompassing molecular, neurobiological, behavioral, and nutritional dimensions. Aging alters the circadian system, particularly melatonin secretion, amplitude, and receptor sensitivity, and these changes affect sleep architecture, metabolism, and cognitive function. Melatonin depicts pleiotropic actions as a circadian synchronizer, sleep facilitator, mitochondrial protector, immunomodulator, and neuroprotective agent. Evidence suggests that age-related melatonin decline contributes to “accelerated” aging through increased oxidative stress, mitochondrial dysfunction, and inflammatory burden (“inflammaging”), whereas restoring melatonin levels, via supplementation or diet, may mitigate such processes. In parallel, we address the emerging field of chrononutrition, emphasizing that the timing of food intake and consumption of melatonin-rich or polyphenol-containing foods can enhance circadian rhythmicity, improve sleep quality, and reduce age-related metabolic and oxidative stress. Melatonin decline represents both a biomarker and mediator of circadian aging. Integrating chronobiological and nutritional interventions, including timed melatonin supplementation, dietary chronobiotics, and lifestyle alignment with the light–dark cycle, holds promise for preserving circadian integrity, delaying physiological aging, and extending health span. Forthcoming research should focus on personalized chronotherapy and nutritional chronobiology to better harness melatonin’s therapeutic potential in aging populations.

## 1 Introduction

Aging is associated with profound alterations in circadian regulation, sleep architecture, metabolic homeostasis, and redox balance. Although these processes are often discussed separately, increasing evidence suggests that they are tightly interconnected through common regulatory mechanisms. Among these, melatonin emerges as a central integrative signal linking circadian timing, sleep regulation, nutritional cues, and antioxidant defense, particularly in the context of aging (Su et al., 2025).

Importantly, aging should not be viewed exclusively as a pathological process. Many age-related changes in circadian rhythms and sleep architecture, such as reduced rhythm amplitude, phase advance, and altered sleep consolidation, may reflect adaptive remodeling of the circadian–homeostatic system rather than intrinsic dysfunction (Scullin and Bliwise, 2015). These modifications can represent physiological adjustments to accumulated environmental exposure, altered metabolic demands, and changes in neuroendocrine sensitivity across the lifespan (Videnovic et al., 2014).

Accordingly, it is essential to distinguish between normal, age-expected circadian adaptation and pathological circadian disruption. While adaptive circadian remodeling may preserve functional stability under changing biological conditions, pathological circadian misalignment is typically associated with disease states, accelerated aging, neurodegeneration, metabolic disorders, or chronic exposure to chronodisruptive factors such as irregular light exposure or feeding patterns (Hood and Amir, 2017).

Melatonin is not only the principal hormonal output of the circadian system but also a pleiotropic molecule with chronobiotic, antioxidant, immunomodulatory, and neuroprotective properties.

In this review, the term “chronobiotic” is used with conceptual differentiation. In its strict sense, chronobiotics refer to agents capable of entraining or phase-shifting circadian rhythms through direct action on the central or peripheral clocks, with melatonin representing the prototypical example (Arendt and Skene, 2005). In a broader descriptive sense, the manuscript also discusses chronobiotic-like or circadian-modulating interventions, including specific dietary components, polyphenols, feeding schedules, and microbiota-related factors, which influence circadian physiology without directly inducing phase shifts (Zawilska et al., 2009).

This distinction is maintained throughout the manuscript to avoid conceptual dilution and to clearly differentiate between true circadian entrainment and indirect modulation of circadian function (Cardinali et al., 2012).

Age-related declines in melatonin secretion and signaling have been consistently associated with circadian rhythm dampening, sleep fragmentation, increased oxidative stress, and chronic low-grade inflammation, all hallmarks of biological aging. Thus, melatonin represents both a biomarker and a mediator of circadian aging (Wu et al., 2024).

The aim of the present narrative review is to provide an integrative synthesis centered on melatonin, examining how its age-related decline mechanistically connects circadian disruption, sleep impairment, chrononutritional factors, and antioxidant defenses. Rather than treating these domains independently, we propose a unifying framework in which melatonin functions as a physiological hub coordinating temporal organization and cellular protection. This perspective allows a more coherent understanding of how lifestyle and nutritional interventions may preserve circadian integrity and promote healthy aging.

Accordingly, this review focuses on mechanistic integration rather than exhaustive coverage of each individual domain (Bocheva et al., 2024; Drăgoi et al., 2019a; Panagiotou et al., 2021; Pévet, 2003; Reiter and Tan, 2002; Stanciu et al., 2019; Van Reeth et al., 2001; Verma et al., 2023a,b) In parallel, emerging evidence suggests that nutritional and lifestyle factors can modulate circadian physiology and melatonin production, opening a new arena termed chrononutrition. Chrononutrition refers to aligning food intake patterns (timing, frequency, and composition of meals) with the body’s circadian clock to optimize metabolic health and sleep-wake rhythms. Disrupted feeding times – such as eating during the biological night or at irregular schedules – can themselves induce circadian misalignment and worsen sleep quality. However, strategic meal timing (e.g., consuming the majority of calories earlier in the day, avoiding late-evening meals) and specific diet composition (e.g., higher tryptophan, lower glycemic load at night) have been shown to produce positive effects on sleep and circadian regulation (Salehi et al., 2019; Tan et al., 2014; Verma et al., 2023a). Certain bioactive food components termed chronobiotics can directly influence clock pathways – for instance, meals rich in the melatonin precursor tryptophan or foods containing melatonin (sometimes termed phytomelatonin) may augment nocturnal melatonin levels and improve sleep in both infants and older adults. Furthermore, many antioxidant nutrients (e.g., vitamins, polyphenols) might protect circadian homeostasis by counteracting oxidative stress, which is known to impair clock function. Given that oxidative damage and chronic inflammation are hallmarks of aging, diet-derived antioxidants – including melatonin itself – could play a pivotal role in promoting healthy aging via maintenance of circadian system integrity (Cardinali et al., 2024; Gruia et al., 2009; Moroşan et al., 2022; Reiter et al., 2024).

In this review, aging refers to the biological aging process, healthy aging denotes the preservation of functional and circadian integrity, and circadian disruption describes pathological alterations or misalignment of circadian rhythms.

**FIGURE 1:**
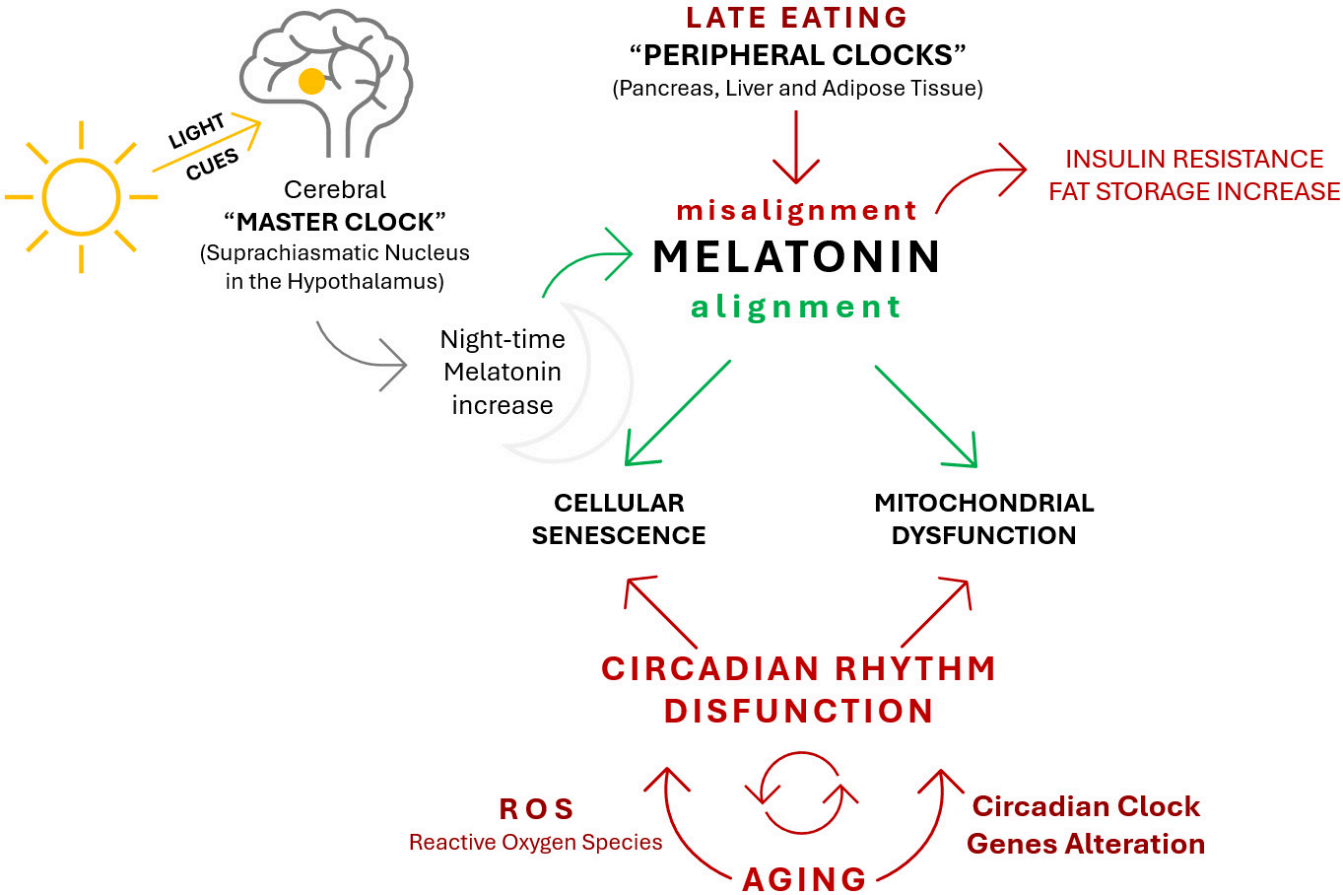
Age-related remodeling of the circadian–sleep system (National Institute of General Medical Sciences [NIGMS], 2020; Reytor-González et al., 2025; Verma et al., 2023a).

Figure 1 illustrates how aging is associated with progressive changes in circadian rhythm amplitude, phase timing, and sleep architecture. It highlights the distinction between adaptive circadian remodeling during normal aging and pathological circadian disruption linked to disease, providing a conceptual framework for understanding age-dependent alterations in sleep–wake regulation.

Light cues entrain the cerebral master clock located in the suprachiasmatic nucleus (SCN) of the hypothalamus, which in turn governs the nocturnal rise in melatonin secretion. Proper night-time melatonin signaling promotes temporal alignment between the central clock and peripheral clocks in metabolically active tissues, including the liver, pancreas, and adipose tissue. In contrast, late eating acts as a potent chronodisruptive cue for peripheral clocks, leading to circadian misalignment that interferes with melatonin-mediated synchronization.

Circadian misalignment is associated with adverse metabolic outcomes, such as increased insulin resistance and enhanced fat storage. Reduced or mistimed melatonin signaling contributes to mitochondrial dysfunction and cellular senescence, thereby weakening cellular resilience. These processes converge on circadian rhythm dysfunction, which is further reinforced by oxidative stress, increased reactive oxygen species (ROS) production, and alterations in circadian clock gene expression. Together, these interconnected pathways establish a self-perpetuating cycle that accelerates biological aging.

In this review, aging is defined as a progressive, organism-level process characterized by gradual functional and systemic changes over time. Cellular senescence refers to a molecular and tissue-level mechanism involving irreversible cell-cycle arrest, altered metabolic activity, and pro-inflammatory signaling. Inflammaging describes the chronic, low-grade, sterile inflammatory state that accompanies aging and contributes to age-related disease susceptibility. These constructs are related but not interchangeable and are addressed at different mechanistic levels throughout the manuscript.

For conceptual clarity, it is important to distinguish between aging, cellular senescence, and inflammaging, as these terms refer to related but distinct biological processes operating at different organizational levels. Aging is defined here as an organism-level, progressive functional and systemic process characterized by gradual changes in physiological resilience and homeostatic regulation across the lifespan (López-Otín et al., 2013). Cellular senescence refers to a molecular and tissue-level mechanism involving irreversible cell-cycle arrest, altered metabolic activity, and the acquisition of a senescence-associated secretory phenotype (SASP), which can influence tissue function and intercellular communication. Inflammaging describes a chronic, low-grade, sterile systemic inflammatory state that emerges with aging and contributes to increased susceptibility to age-related diseases (Campisi, 2011; Franceschi et al., 2017).

Throughout this review, these constructs are addressed according to the level of evidence discussed: organismal aging in relation to circadian rhythm alterations and sleep architecture; cellular senescence in the context of oxidative stress, mitochondrial dysfunction, and clock gene dysregulation; and inflammaging when considering chronic inflammatory signaling, immune–metabolic interactions, and age-related disease risk.

Given the multidisciplinary nature of circadian and aging research, it is essential to distinguish between different levels of evidence. Throughout this review, mechanistic insights derived from cellular and animal models are discussed separately from translational observations and human clinical or observational data. Where direct clinical evidence is limited, mechanistic findings are interpreted as hypothesis-generating rather than causal, in order to avoid overextension of preclinical results.

## 2 The circadian system, sleep, and their changes with aging

This section outlines the fundamental organization of the circadian system and describes how circadian rhythms and sleep architecture are modified across the aging process. These age-related changes provide the biological framework necessary to understand subsequent alterations in melatonin signaling, metabolic regulation, and redox homeostasis.

The circadian timing system in mammals is organized in a hierarchical manner with the SCN in the hypothalamus serving as the master clock. The SCN consists of a bilaterally paired cluster of ∼20,000 neurons that generate self-sustained oscillations of gene expression and electrical activity with a period of about 24 h. The SCN receives direct light input from intrinsically photosensitive retinal ganglion cells in the eye, which entrains the clock to the external 24-h light/dark cycle each day. In turn, the SCN synchronizes subsidiary circadian oscillators present in virtually every peripheral tissue (heart, liver, kidney, lungs, immune cells, etc.) coordinating their rhythms via neural and hormonal signals. This multi-oscillator system allows physiological processes to align with the appropriate time of day – promoting activity, feeding, and catabolic processes during the biological daytime, and rest, fasting, and cellular repair during the biological night. A properly synchronized circadian system thereby preserves internal temporal order and optimizes health (Drăgoi et al., 2024c; Verma et al., 2023a).

**FIGURE 2:**
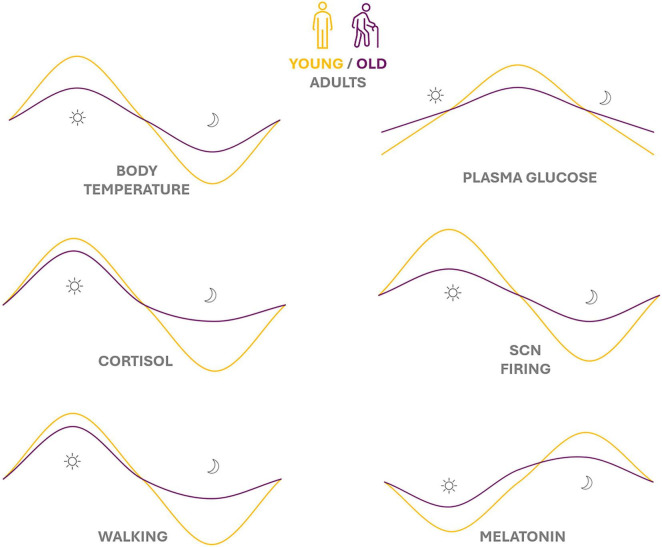
Melatonin as a central integrative signal in circadian aging. [Figure concept adapted from Hood and Amir (2017)].

Aging, however, imposes significant stress on the circadian system. In humans, many circadian-regulated rhythms exhibit attenuated amplitude and phase shifts in older age (Hood and Amir, 2017; Panagiotou et al., 2021). Figure 2 illustrates schematically some documented changes: rhythms of locomotor activity (rest-activity cycle), core body temperature, hormone secretion (e.g., melatonin at night, cortisol in the morning), SCN neuronal firing rate, and even daily metabolic indicators like plasma glucose all show diminished oscillation amplitudes in older adults compared to younger adults. In some cases, the timing (peak phase) of rhythms is advanced with age, occurring earlier in the 24-h cycle. These alterations manifest behaviorally as earlier wake times and bedtimes, the classic “advanced sleep phase” tendency of the elderly, and fragmented, less robust daily patterns of activity, body temperature, and hormone levels. Age-related deterioration of the central SCN clock has been demonstrated in animal models at molecular and electrophysiological levels (e.g., reduced amplitude of SCN clock gene expression and firing) and likely underlies some of these changes. Compounding this, age-associated risk factors – such as neurodegenerative changes, ophthalmologic changes reducing light input, sedentary lifestyle, and irregular environmental cues – can all contribute to circadian dysregulation in the elderly.

Figure 2 depicts melatonin as a central physiological hub linking the circadian clock, sleep regulation, and redox homeostasis. It emphasizes how age-related declines in melatonin secretion and signaling contribute to circadian dampening, sleep fragmentation, increased oxidative stress, and chronic low-grade inflammation.

Conceptual schematic illustrating the attenuation of circadian rhythm amplitude and phase shifts in aging, with a particular emphasis on nocturnal melatonin decline as a central driver of sleep fragmentation, metabolic dysregulation, and reduced antioxidant capacity (Hood and Amir, 2017).

The figure highlights age-related changes in key physiological variables, including rest–activity cycles, core body temperature, hormonal secretion (melatonin and cortisol), suprachiasmatic nucleus (SCN) neuronal activity, and metabolic indicators such as glucose regulation.

In younger adults, these variables exhibit robust, high-amplitude circadian oscillations, whereas in older individuals circadian rhythms become dampened and may show phase advances. The decline in nocturnal melatonin secretion is depicted as a central integrative mechanism contributing to sleep fragmentation, metabolic dysregulation, and reduced antioxidant capacity.

The figure is conceptual in nature and was developed by the authors based on previously published literature on circadian aging.

These age-related circadian changes should not be uniformly interpreted as dysfunction. In healthy aging, attenuated circadian amplitude and phase shifts may represent adaptive recalibration of the circadian system, optimizing energy expenditure, sleep–wake timing, and metabolic processes under age-related physiological constraints. Such adaptations may become maladaptive only when compounded by chronic circadian misalignment, neurodegenerative pathology, or persistent environmental disruption.

In healthy young adults (yellow curves), robust high-amplitude circadian rhythms are evident in diverse physiological variables (activity levels, core body temperature, SCN firing rate, hormonal secretion such as melatonin and cortisol, glucose metabolism, etc.) across the 24-h day. In older adults (purple curves), many of these rhythms show a decrease in amplitude, indicating weaker circadian oscillation. Some rhythms also exhibit a phase advance (earlier timing of peak) in aging. These age-related changes can lead to fragmented sleep-wake patterns, blunting of daily metabolic and hormonal cycles, and an overall loss of internal synchrony. Such circadian dampening and phase shifts are thought to contribute to health issues in the elderly. One of the most visible outcomes of circadian disruption in aging is sleep disturbance. Normal aging is associated with reduced sleep duration, lighter and more fragmented sleep, increased nocturnal awakenings, and a shift toward earlier wake times. These changes correlate with the weakened circadian signals (for instance, a dampened melatonin rhythm and blunted nighttime drop in body temperature) that normally consolidate sleep at night. It has been shown that prolonged disturbances of circadian rhythms and sleep are detrimental to multiple aspects of health at any age, but older individuals may be especially vulnerable. Chronic sleep insufficiency and rhythm disruption in the elderly can exacerbate metabolic syndrome (hypertension, insulin resistance, dyslipidemia) and promote neuropsychiatric problems (Panagiotou et al., 2021; Touitou et al., 2017). Moreover, epidemiological studies link poor sleep in older adults to increased risks of cognitive impairment and Alzheimer’s disease, impaired immune function, and even higher mortality. Notably, recent research suggests this relationship is bi-directional: aging causes circadian/sleep disruption, and conversely, circadian disruption can accelerate aspects of aging (Acosta-Rodríguez et al., 2021).

Given these insights, there is intense interest in identifying interventions that can stabilize or strengthen circadian rhythms in older people. Lifestyle regularity is one important factor – maintaining consistent daily schedules of light exposure, activity, and meals helps entrain the aging clock. In particular, appropriately timed light exposure is a powerful synchronizer for the SCN: morning daylight can help anchor the phase of the circadian clock, counteracting the tendency for phase advance, while minimizing bright light at night, especially blue-enriched light from screens, prevents melatonin suppression and preserves sleep quality (Brown et al., 2022; Voiculescu et al., 2016). Alongside light, nutrition and meal timing have emerged as crucial modulators of circadian function. Feeding behavior is itself under circadian control, with appetite, digestion, and metabolic hormone release peaking at certain times, but reciprocally, the timing of food intake also feeds back onto the clock as a zeitgeber for peripheral organs. Irregular meal times or eating during the biological night can desynchronize peripheral clocks from the SCN, contributing to internal misalignment. In fact, shift workers who consume large meals at night show altered circadian gene expression and gut microbiome rhythms, leading to metabolic disturbances. In contrast, aligning meals to appropriate times during daytime and fasting at night reinforces the normal day-night metabolic rhythm. For example, studies indicate that time-restricted feeding – limiting food intake to a ∼8–12 h window during the active phase – can improve circadian rhythm robustness and metabolic health markers in animals and humans. Thus, behavioral interventions as light and meal scheduling, exercise timing, etc., offer feasible strategies to support the aging circadian clock (Hernández-García et al., 2020; Zuraikat et al., 2021).

Crucially, melatonin lies at the intersection of many of these processes. As the hormonal effector of the circadian system, melatonin plays a multifaceted role in coordinating sleep and other rhythms (Pereira et al., 2022).

Circadian alterations observed during aging exist along a continuum ranging from adaptive reorganization to maladaptive disruption. Pathological outcomes are more likely to emerge when adaptive mechanisms are overwhelmed by chronic stressors, persistent chronodisruption, or underlying disease processes.

Importantly, many of the age-related alterations described above converge on a common endocrine signal: melatonin. As the primary hormonal mediator of circadian night, melatonin plays a pivotal role in stabilizing sleep–wake rhythms and synchronizing central and peripheral clocks. Its decline with aging represents a key mechanistic link between circadian disruption and downstream metabolic and oxidative consequences, as discussed in the following section.

Standardize all references to rhythm impairment as “circadian disruption,” unless a specific subtype (e.g., phase advance, amplitude dampening) is discussed.

## 3 Melatonin: physiology and pleiotropic roles in aging

Building on the age-related changes in circadian regulation, this section focuses on melatonin as a central integrative signal linking circadian timing, sleep regulation, and antioxidant defense. Particular emphasis is placed on how age-associated declines in melatonin contribute to circadian dampening and impaired cellular resilience.

Evidence discussed in this section is derived primarily from preclinical and mechanistic studies, including cellular systems and animal models, and should therefore be interpreted in a mechanistic rather than clinical context.

Age-related changes in melatonin secretion, circadian rhythms, and physiological function should not be interpreted as a simple linear causal chain. Rather, these processes interact through complex, multicausal pathways involving bidirectional feedback loops between circadian regulation, metabolic status, inflammatory signaling, and environmental exposures.

Melatonin Synthesis and Circadian Function: Melatonin (chemically, N-acetyl-5-methoxytryptamine) is an indoleamine synthesized from tryptophan in the pineal gland during darkness. In humans, melatonin production is low during the day and begins to rise in the evening, approximately 2 h before habitual bedtime, when the SCN clock signals the onset of night. Pineal melatonin levels then surge to a peak in the middle of the night (around 2–4 a.m. for a typical schedule), and fall rapidly toward morning as light inhibits further synthesis. The duration of melatonin secretion at night encodes night length (photoperiod), longer winter nights allowing a more prolonged melatonin signal than short summer nights. This melatonin rhythm is often called the “darkness hormone” signal; it helps organize the body’s internal physiology to the environmental day/night cycle (Drăgoi et al., 2024d; Zarezadeh et al., 2022).

Importantly, the physiological effects of melatonin are determined not only by dosage but also by the timing of administration. Timing-dependent effects primarily influence circadian phase alignment, sleep–wake timing, and synchronization between central and peripheral clocks, whereas dose-dependent effects are more closely related to signal amplitude, antioxidant capacity, and cytoprotective actions. Distinguishing between these temporal and quantitative dimensions is critical for interpreting melatonin’s role in circadian regulation and aging.

Melatonin’s primary function is as a circadian synchronizer or “chronobiotic.” It reinforces the 24-h rhythm throughout the body by coordinating physiological activities to night versus day. At night, rising melatonin acts on MT1/MT2 receptors in various tissues to induce nighttime physiology: it promotes the onset of sleep, lowers core body temperature, slows gastrointestinal activity, and modulates metabolism to a nocturnal state. Perhaps most importantly, melatonin feeds back onto the master clock, the SCN itself is rich in melatonin receptors, and melatonin binding can shift the timing of the clock’s firing rhythms. Melatonin taken exogenously in the afternoon or early evening can advance the circadian phase, shifting the rhythm earlier, which is why it is used to treat circadian phase disorders like jet lag or advanced sleep phase in elderly. Conversely, melatonin in the morning can delay the clock. Through such effects, melatonin is considered a prototypical chronobiotic agent, adjusting the timing of the internal clock. Endogenous melatonin secretion each night essentially acts as a temporal synchronizing signal that helps keep peripheral clocks in phase with the central SCN clock during the rest period. In aging, when melatonin output diminishes, this synchronization is weakened – contributing to internal desynchrony and various functional impairments (Dufoo-Hurtado et al., 2020).

To summarize melatonin’s roles relevant to aging, Table 1 provides an overview of some key functions of melatonin and how they change or impact physiology in older adults. Melatonin is truly a pleiotropic hormone: beyond circadian rhythm regulation and sleep, it has notable antioxidant, immunological, and neuroprotective effects – all of which have implications for age-related degeneration.

**TABLE 1:** Major roles of melatonin and their relevance to aging.

Role of melatonin	Mechanism/effect	Relevance to aging
Circadian synchronizer	Endogenous “darkness” signal that entrains internal 24-h rhythms; melatonin release at night orchestrates sleep-wake timing, core body temperature, hormonal cycles (Verma et al., 2023b)	Melatonin secretion declines with age, and the circadian rhythm is altered. A weaker melatonin rhythm in older adults leads to reduced circadian signal strength and internal misalignment. Enhancing melatonin by means of supplementation or lifestyle may help resynchronize circadian rhythms in the elderly (Verma et al., 2023a,b).
Sleep promoter	Facilitates sleep onset and maintenance by acting on melatonin receptors (MT_1_/MT_2_) in the SCN and elsewhere to induce sedation and lower nocturnal alertness. Also slightly reduces core body temperature, which aids sleep initiation (Panagiotou et al., 2021; Touitou et al., 2017).	Melatonin’s sleep-facilitating effect becomes critical as insomnia prevalence rises in older adults. Diminished melatonin in aging is linked to difficulty falling asleep and fragmented sleep. Clinical trials show that timed melatonin (or analogues like ramelteon) in elderly patients modestly improve sleep latency, total sleep time, and sleep quality, underscoring melatonin’s therapeutic potential for age-related insomnia (Touitou et al., 2017).
Antioxidant defender	Potent free radical scavenger and broad-spectrum antioxidant. Melatonin is amphiphilic and easily enters all cellular compartments, including crossing the blood-brain barrier. It directly neutralizes reactive oxygen and nitrogen species and upregulates antioxidant enzymes (e.g., glutathione peroxidase, superoxide dismutase). Notably, melatonin concentrates in mitochondria, reducing oxidative damage at its source (Drăgoi et al., 2012a,b; Mo et al., 2024; Reiter et al., 2024; Zarezadeh et al., 2022).	Aging is characterized by increased oxidative stress and declining endogenous antioxidant capacity. Melatonin levels drop in parallel with the age-related decline in total antioxidant capacity. This loss may exacerbate mitochondrial DNA damage and cellular oxidative injury in aging. Restoring melatonin (through supplements or diet) in aged animals reduces oxidative damage and improves mitochondrial function, suggesting a geroprotective effect. Melatonin’s antioxidant role thus shields cells (including clock neurons) from free-radical damage that accumulates with age, potentially slowing aspects of the aging process (Drăgoi et al., 2019a; Zeng et al., 2021).
Immune modulator and anti-inflammatory	Modulates immune cell activity and cytokine production. Melatonin tends to suppress pro-inflammatory mediators (e.g., inhibits NF-κB activation and reduces release of TNF-α, IL-6) while enhancing anti-inflammatory cytokines. It also influences the circadian rhythm of immune parameters, many immune cells having melatonin receptors (Fu et al., 2025; Hardeland, 2018).	The elderly often exhibit chronic low-grade inflammation (“inflammaging”) characterized by elevated pro-inflammatory cytokines (Hardeland et al., 2015). A decline in melatonin’s immunomodulatory influence with age may permit an exaggerated inflammatory baseline. Indeed, melatonin loss is associated with higher nocturnal cytokine levels in aging. Studies indicate melatonin supplementation can attenuate inflammatory responses in aged animals and humans, potentially counteracting age-related inflammatory conditions. For example, melatonin has shown benefit in reducing neuroinflammation in models of cognitive decline. By restraining excessive inflammation, melatonin might mitigate tissue damage in aging and diseases of aging (e.g., arthritis, neurodegeneration) (Fernández-Ortiz et al., 2022; Hardeland, 2018; Mathada and Somappa, 2022).
Neuroprotective agent	Guards neurons against excitotoxicity and apoptosis; stabilizes mitochondrial function in neurons and reduces oxidative neuronal damage. Melatonin can decrease accumulation of β-amyloid and hyperphosphorylated tau in experimental models, and it improves synaptic plasticity and memory in aging rodent studies. Also exerts sedative and anti-excitatory effects that calm neural networks (Cardinali, 2019; Nicolae et al., 2013; Reiter et al., 2024).	The age-related melatonin decline may contribute to increased neuronal vulnerability and brain aging. Higher oxidative stress and poorer sleep (due to low melatonin) can accelerate neurodegenerative processes. In animal models of Alzheimer’s and Parkinson’s disease, melatonin treatment reduces neuronal loss, preserves synapses, and slows cognitive decline (Martín Giménez et al., 2022; Pévet, 2003). Some clinical trials in patients with mild cognitive impairment suggest melatonin might help preserve cognitive function, though large-scale evidence is pending. Melatonin’s multi-modal neuroprotection (antioxidant, anti-excitotoxic, anti-amyloid) positions it as a promising candidate to mitigate age-related neurodegeneration and cognitive impairment (Hu et al., 2023; Lv et al., 2024; Sumsuzzman et al., 2021; Terao and Kodama, 2024; Zhang et al., 2025).

Melatonin’s decline in aging has widespread implications – weakening circadian coordination, diminishing sleep quality, lowering antioxidant defense, increasing inflammation, and reducing neuroprotection, all of which may contribute to “accelerated” aging and age-related diseases. Conversely, maintaining or restoring melatonin levels in older individuals may help preserve circadian robustness, sleep health, and cellular homeostasis, thereby promoting healthier aging.

Melatonin may support circadian robustness by preserving rhythm amplitude, phase stability, and sleep consolidation during aging.

Declining melatonin levels may contribute to reduced circadian signal robustness; however, whether these changes reflect adaptive remodeling or maladaptive circadian disruption depends on the broader physiological and environmental context.

Age-related decline in melatonin secretion may contribute to reduced circadian signal robustness. While this decline can be part of normal adaptive aging, it may become clinically relevant when combined with chronic circadian disruption, neurodegenerative processes, metabolic disease, or persistent oxidative and inflammatory stress.

Use “healthy aging” explicitly when discussing protective, preventive, or resilience-promoting effects of melatonin and chrononutrition.

## 4 Chrononutrition: timing of food intake and the circadian clock

This section shifts the focus from endogenous circadian regulation to modifiable lifestyle factors, examining how meal timing, dietary composition, and specific bioactive nutrients interact with circadian clocks and melatonin physiology. The concept of chrononutrition is discussed as a practical interface between behavior, metabolism, and circadian health.

Within this melatonin-centered framework, chrononutrition represents a critical modulatory pathway through which nutritional timing and composition can influence melatonin secretion, circadian alignment, and sleep quality.

Where applicable, the strength and type of evidence supporting the discussed interventions are explicitly indicated throughout the manuscript. Preclinical findings derived from cellular and animal models are distinguished from translational observations and human data, including pilot studies, observational cohorts, and randomized clinical trials. In addition, reported outcomes are contextualized according to their nature, whether subjective (e.g., sleep quality, self-reported symptoms), physiological (e.g., circadian phase markers, metabolic parameters), or biochemical (e.g., oxidative stress markers, inflammatory mediators). This approach aims to improve transparency and to facilitate appropriate interpretation of the evidence base.

It is increasingly evident that when we eat may be just as important as what we eat for maintaining metabolic health and synchronized circadian rhythms. The human body’s metabolic processes have intrinsic daily oscillations: glucose tolerance, insulin sensitivity, digestive enzymes’ activity, and hunger hormones all vary by time of day under circadian control (Drăgoi et al., 2019b; Pereira et al., 2022). Eating at suboptimal times, such as late at night, can therefore result in misalignment between food intake and the body’s metabolic readiness, leading to poor digestion and metabolic stress. One study noted that consuming a high-glycemic-index carbohydrate meal 4 h before bedtime significantly shortened sleep onset latency, making it easier to fall asleep compared to eating the same meal 1 h before bed. This presumably is because the earlier timing allows adequate digestion and promotes tryptophan uptake into the brain well before sleep, facilitating melatonin and serotonin synthesis. In contrast, eating too close to bedtime can cause gastrointestional discomfort and suppress melatonin, as insulin and other postprandial changes can modulate melatonin release, thus impairing sleep (Zuraikat et al., 2021).

Older adults often experience changes in appetite and meal patterns, as earlier dinner times or reduced caloric intake at night. Aligning these changes with circadian biology could be beneficial. Meal timing recommendations for healthy aging usually include: concentrating calorie intake in daytime hours, avoiding large meals late in the evening, and establishing consistent daily meal times. Such practices reinforce the metabolic clock’s alignment with the light/dark cycle. These observations should be considered hypothesis-generating and warrant further investigation in well-designed longitudinal and interventional human studies.

Chrononutritional strategies, including time-restricted feeding (TRF), are discussed primarily as lifestyle interventions designed to enhance circadian alignment and metabolic resilience in the general population and during aging. Evidence supporting their therapeutic application in specific clinical populations such as individuals with metabolic syndrome or type 2 diabetes is considered separately and interpreted with caution.

Research on TRF in older rodents has shown improved circadian gene expression and reduced age-related weight gain and inflammation, suggesting TRF could combat some aging processes. Although long-term TRF trials in the elderly are still limited, short-term studies in adults indicate benefits like better glucose control and blood pressure when eating windows are aligned with daytime (Panagiotou et al., 2021; Pattnaik et al., 2022).

In the context of chrononutrition, the timing of food intake appears to exert stronger circadian effects than nutrient quantity alone. While dietary composition and dose influence metabolic load and antioxidant availability, meal timing primarily acts as a zeitgeber for peripheral clocks, thereby modulating circadian alignment independently of caloric intake.

Another critical link between nutrition timing and circadian health involves the gut microbiome. The intestinal microbiome exhibits its own daily rhythmicity in composition and function, which is entrained by feeding cycles. During the day (active phase), certain microbial populations flourish to aid digestion, whereas at night (fasting phase) the microbiome shifts.

Microbiota-targeted strategies are considered within a lifestyle and nutritional modulation framework for healthy aging, while their potential role as therapeutic adjuncts is discussed in relation to specific clinical conditions, such as metabolic or inflammatory disorders, where emerging but still limited evidence is available. Disruption of normal feeding-fasting cycles (e.g., due to erratic eating schedules or late-night eating) causes microbiota dysbiosis and loss of these rhythms. In turn, an imbalanced microbiome can negatively affect the host’s circadian clock and metabolism (Matenchuk et al., 2020; Wang et al., 2023). Notably, experiments in mice showed that a high-fat diet or clock-disrupting conditions alter microbial rhythms, which then contributes to hepatic circadian clock dysfunction and obesity. On the other hand, prebiotic fibers that promote beneficial gut microbes can enhance SCFA (short-chain fatty acid) production in a rhythmic manner; these microbial metabolites, as butyrate, can act as signals to peripheral clocks and help maintain rhythm synchrony. For example, oral administration of fiber or SCFAs in mice shifted peripheral clock gene phases in a time-dependent way, implying that dietary fibers could be used to correct circadian misalignment via the microbiome. In a human trial, high-fiber diets improved sleep and even shifted chronotype earlier (toward “morningness”), potentially by these gut-mediated pathways (Drăgoi et al., 2025; Tarta-Arsene et al., 2017; Wang et al., 2022; Yang et al., 2023).

Importantly, responses to melatonin supplementation and chrononutritional strategies exhibit substantial inter-individual variability among older adults. Factors such as chronotype, comorbidities, sex-specific differences, light exposure patterns, frailty status, and polypharmacy can significantly influence circadian responsiveness and metabolic outcomes. Age-related changes in drug metabolism, altered light sensitivity, and interactions with commonly prescribed medications further contribute to heterogeneous responses. Acknowledging this variability is essential for interpreting existing evidence and underscores the need for individualized approaches when applying circadian and nutritional interventions in aging populations.

Chrononutritional strategies may contribute to improved metabolic alignment and circadian coherence.

In summary, chrononutrition approaches for older individuals emphasize synchronizing meals with circadian rhythms: eat during daylight and fast at night, maintain regular meal schedules, and incorporate foods that support circadian signals, rich in melatonin or its precursors. This not only benefits sleep but also can attenuate age-related metabolic disturbances.

## 5 Dietary chronobiotics and antioxidants: nutritional interventions for healthy aging

This section explores oxidative stress as a key mechanistic link between circadian disruption and aging-related cellular dysfunction. It highlights the bidirectional interactions between redox imbalance, mitochondrial function, and circadian clock regulation, with particular attention to the antioxidant role of melatonin.

A variety of bioactive food components have been identified as potential chronobiotics – substances that can modulate circadian rhythms. Perhaps the most direct chronobiotic from diet is melatonin itself.

In this manuscript, the term “chronobiotic” is used primarily in a functional sense, referring to agents capable of modulating circadian physiology, while acknowledging that only a subset (such as melatonin) fulfills the strict definition of circadian entrainment or phase-shifting agents.

Melatonin is not only produced in animals; it is also found in many plants and foods, sometimes termed phytomelatonin when from plant sources. Examples of melatonin-containing foods include certain fruits, especially tart cherries, grapes, bananas, pineapples, vegetables, grains, nuts and seeds, as well as animal products like milk (Pereira et al., 2022; Salehi et al., 2019; Tan et al., 2014). Although the concentrations are much lower than pharmacological supplement doses, dietary melatonin can be bioavailable and may contribute to circulating melatonin levels. It has been theorized that consuming melatonin-rich foods in the evening could raise nocturnal melatonin and enhance sleep quality, providing a nutritional alternative or adjunct to melatonin supplements. Indeed, small human studies found that eating foods like pineapples, kiwis, oranges, or bananas, all containing relevant levels of melatonin, increased serum melatonin by ∼ melatonin 2–3-fold shortly after consumption (Doherty et al., 2023; Pereira et al., 2022; Salehi et al., 2019; Tan et al., 2014). Importantly, these foods also tended to increase total antioxidant capacity in the bloodstream, reflecting melatonin’s antioxidant contribution. Diets high in fruits and vegetables (some of which contain melatonin and related indolic compounds) are associated with better sleep in epidemiological studies, though causality is hard to prove. Nonetheless, such findings have led researchers to consider melatonin-rich foods as “nutraceuticals” for sleep and circadian health (Matenchuk et al., 2020).

Among melatonin-rich foods, tart cherries have gained particular attention. Montmorency tart cherries and certain sweet cherry cultivars contain measurable levels of melatonin (in the range of 0.01–0.2 ng per gram of fresh weight, varying by variety) (Kimble et al., 2022). A seminal randomized trial by Garrido et al. (2010) 2012 had middle-aged and elderly participants consume a Jerte Valley cherry concentrate for several weeks: the results showed improved sleep quality and duration, a shift toward a more normal sleep-wake rhythm, and significant increases in urinary 6-sulfatoxymelatonin, a metabolite of melatonin, as well as total antioxidant capacity. In fact, nocturnal rest improved and nighttime melatonin metabolite excretion increased ∼3-fold in those on the cherry-enriched diet compared to controls. The rise in antioxidant status was attributed to melatonin and serotonin in the cherries and possibly other polyphenols. This study and subsequent trials confirm that tart cherry juice or concentrate supplementation in older adults can modestly benefit insomnia – reducing time awake after sleep onset and increasing total sleep time – while also reducing inflammatory markers. Thus, tart cherries act as a natural chronobiotic and antioxidant cocktail, countering the melatonin deficit and oxidative stress of aging (Chai et al., 2019; Delgado et al., 2013; Garrido et al., 2010, 2012; Sinclair et al., 2021).

Another fruit with sleep-promoting, antioxidant properties is kiwifruit. Kiwis are not particularly high in melatonin, though they contain some, but they are an exceptionally rich source of serotonin as well as vitamins C and E and folate. Serotonin is a precursor to melatonin and has its own role in modulating sleep-wake cycles. In a trial of adults with chronic sleep difficulties, consuming two kiwifruits 1 h before bedtime each night for 4 weeks led to significant improvements in sleep onset, duration, and efficiency. Participants fell asleep faster and had fewer awakenings, with total sleep time increasing by 13%. The authors speculated that kiwifruit’s high antioxidant content might reduce oxidative stress that impairs sleep regulation, and the serotonin/folate could aid neurotransmitter balance for sleep. Kiwis also do not require cooking (folate is destroyed by heat), so their nutrients remain intact. While not a direct melatonin chronobiotic, kiwifruit seems to exert sleep benefits via a nutritional synergy of antioxidants and serotonin support, and it is a simple dietary addition that may be especially beneficial for older adults who often have insomnia and low antioxidant intake (Doherty et al., 2023; Lin et al., 2011).

Beyond fruits, milk is notable: cow’s milk obtained from nighttime milking has a higher melatonin content (“night milk”) compared to daytime milk, since cows, like humans, secrete melatonin at night. This melatonin-enriched night milk has demonstrated sleep-enhancing effects in studies, including shortened sleep latency and improved subjective sleep quality. One systematic review concluded that milk and dairy products in general (apart from their melatonin, also containing tryptophan, magnesium, etc.) have a positive impact on sleep, especially in individuals with poor sleep. Some commercially available milk products are marketed as “night milk” or with added tryptophan to capitalize on these findings. While the effects are modest, a warm glass of milk in the evening (a traditional remedy) may indeed have a biochemical basis in promoting sleep in older folks – via both melatonin content and calcium’s role in calming nerves and facilitating tryptophan’s conversion to melatonin (Milagres et al., 2014; Romanini et al., 2019).

Tryptophan itself is an essential amino acid that is the precursor for serotonin and melatonin (Nicolae et al., 2015; Won et al., 2021). Dietary tryptophan availability can influence melatonin synthesis in the pineal. Researchers have experimented with tryptophan-enriched cereals and meals given at night. One study in elderly insomniacs provided a tryptophan-enriched cereal bar in the late evening and found significant improvements in sleep (longer sleep time, fewer awakenings) along with increased melatonin and serotonin metabolite levels in urine. In that trial, morning alertness and wellbeing also improved, presumably due to better sleep and perhaps direct effects of serotonin. Similarly, adding tryptophan-rich ingredients (or tryptophan, adenosine-5’-phosphate, and uridine-5’-phosphate) to infants’ evening meals has been shown to help consolidate infant sleep-wake rhythms. All these point to the utility of boosting tryptophan intake at dinner or bedtime to naturally raise melatonin production at night. Foods high in tryptophan include milk, turkey, chicken, eggs, seeds, and whole grains – so a light snack containing these (e.g., whole-grain cereal with milk) in the evening might aid sleep onset in older people. However, one must balance this with not eating too heavily or too late at night (Bravo et al., 2013; Garrido et al., 2012).

Dietary polyphenols and antioxidant-rich foods are primarily addressed as components of dietary patterns that support physiological resilience and redox balance during aging. Their potential therapeutic relevance in disease-specific contexts is discussed based on preclinical and observational data and should be regarded as hypothesis-generating rather than evidence of clinical efficacy.

A class of dietary chronobiotics receiving growing attention is polyphenols – plant-derived antioxidant compounds – which may impact the molecular clock (Kimble et al., 2022). For instance, nobiletin, a citrus flavonoid, can bind ROR nuclear receptors and has been shown to enhance circadian amplitude and improve metabolic syndrome in mice by acting on clock gene regulation (He et al., 2016). Green tea catechins (like EGCG) and grape seed proanthocyanidins have been reported to influence clock gene expression in peripheral tissues and the SCN. In a mouse model of circadian disruption (constant darkness akin to shift work), supplementation with green tea polyphenols restored normal daily oscillations of glucose metabolism genes and improved insulin sensitivity. Another study found that EGCG (the major green tea catechin) given to mice on a high-fat/high-fructose “western” diet could ameliorate insulin resistance and realign dysregulated liver clock genes (Mi et al., 2017; Qi et al., 2017; Sur and Panda, 2017). Resveratrol, a polyphenol in grapes and red wine, was shown to reduce body weight gain in high-fat-fed rodents and restore rhythmic oscillation of leptin and insulin, essentially re-synchronizing metabolic hormones that had lost clear rhythms due to diet-induced obesity. These nutraceuticals likely act through multiple pathways – as antioxidants reducing cellular stress in clock-regulating tissues, as ligands for clock-modulating receptors, and by influencing sirtuins and other clock-linked metabolic sensors (Ghiţă et al., 2021; Sun et al., 2015). While human evidence is still emerging, foods rich in polyphenols (fruits, vegetables, coffee, cocoa, tea, herbs) are broadly associated with lower inflammation and better metabolic health in aging. It is tantalizing to think some of their benefits may come from reinforcing circadian homeostasis. At minimum, their antioxidant properties complement melatonin’s actions in neutralizing the oxidative stress that accumulates with age and circadian misalignment (Dufoo-Hurtado et al., 2020; Menczel Schrire et al., 2024).

To illustrate some of these findings, Table 2 provides a few examples of nutritional interventions studied for their effects on melatonin levels, sleep quality, and circadian-related outcomes in middle-aged or elderly populations:

**TABLE 2:** Selected nutritional interventions as chronobiotics in aging.

Intervention (nutrient/food)	Study population	Effects on melatonin/sleep/circadian	Source
Tart cherry juice or concentrate	Older adults (50–70 s), insomniacs	↑ Nocturnal melatonin metabolite (6-sulfatoxymelatonin) in urine; ↑ total antioxidant capacity; improved sleep duration and efficiency; reduced night-time awakenings. Cherry varieties highest in phytomelatonin yielded the greatest sleep benefits.	Garrido et al., 2010, 2012
Kiwifruit (2 kiwis before bed)	Adults with sleep disturbances	↓ Sleep onset latency, ↓ awakenings; ↑ sleep time and efficiency after 4 weeks of nightly kiwi consumption. Proposed mechanisms: high antioxidant vitamins C/E reduce oxidative stress, kiwi’s serotonin content aids sleep regulation.	Doherty et al., 2023
Night-time milk (high melatonin)	Elderly individuals with insomnia	Mild improvement in sleep quality metrics (shorter time to fall asleep, longer REM sleep proportion) versus regular milk. Milk taken ∼1 h before bed. Mechanism: ∼10-fold higher melatonin content in night milk plus peptides that promote relaxation.	(Kitano et al., 2014; Komada et al., 2020; Romanini et al., 2019; Sahathevan et al., 2018, 2021; Yamamura et al., 2009)
Tryptophan-enriched cereal snack	Older adults (65+ years) with insomnia	↑ Urinary melatonin (+50%) and serotonin metabolite; ↑ total antioxidant status; self-reported better sleep onset and mood. Objective sleep recordings showed longer sleep time and fewer wake bouts. Mood improvements (less depression/anxiety) also noted, likely via serotonin pathways.	Bravo et al., 2013
Time-restricted feeding (TRF) (8–10h daytime feeding window)	Middle-aged adults (health trial)	Improved alignment of feeding with endogenous metabolic rhythms: ↓ evening appetite swings; some evidence of improved sleep continuity. In animal models, TRF in older mice restored circadian oscillation of clock genes in liver and muscle, and reduced body weight and inflammation independent of calorie intake.	Manoogian et al., 2024; Wilkinson et al., 2020, 2023
Spent coffee ground fiber (SCG) added to diet	Middle-aged adults (pilot study, 3 weeks)	↑ Plasma melatonin levels; shifted chronotype from “evening” toward “morning” type (earlier wake/sleep times) after 21 days of SCG-fortified biscuits daily. Also reported better subjective sleep quality. SCG (rich in prebiotic fiber and phytochemicals) likely acted via improved microbiota and perhaps direct melatonin-like compounds.	Oseguera-Castro et al., 2019

These are a few examples of how targeted nutritional interventions can influence melatonin production, circadian timing, and sleep parameters, particularly in middle-aged and older adults. These studies support the concept of chrononutrition – dietary choices (both content and timing) can serve as adjunct therapies to strengthen circadian rhythms and ameliorate age-related sleep/circadian disturbances (Margina and Drăgoi, 2023). Notably, interventions like tart cherries and TRF not only improved sleep or rhythm markers but also enhanced antioxidant status and metabolic measures, suggesting holistic benefits for healthy aging.

It is evident from the above that diet and nutrients offer promising tools to combat circadian and sleep disturbances in aging. They can be used alongside other behavioral strategies, as exercise, light therapy and medical treatments. For instance, exercise is another potent zeitgeber that can improve sleep and circadian alignment in older adults, as regular moderate aerobic exercise has been shown to increase nocturnal melatonin output and improve sleep efficiency in the elderly. When combined with dietary chronotherapy – e.g., morning exercise plus high-protein breakfast, and avoidance of caffeine or heavy meals late in day – the synergistic effect can be substantial in restoring a robust 24-h rhythm (Dumitrescu et al., 2025; Drăgoi et al., 2025; Panagiotou et al., 2021).

These interventions may support functional resilience and circadian regulation under age-related physiological constraints.

Here, timing-related effects predominantly affect circadian coordination and mitochondrial rhythmicity, whereas dose-related effects contribute to redox buffering capacity and attenuation of oxidative damage.

## 6 Antioxidative and anti-aging effects of melatonin

The antioxidative and anti-aging effects discussed in this section should be viewed primarily through the lens of melatonin biology. As both an endogenous antioxidant and a regulator of mitochondrial function, melatonin uniquely bridges circadian regulation and redox homeostasis, positioning oxidative stress as a downstream consequence of circadian and melatonergic decline in aging. One recurring theme is the role of oxidative stress in aging and how melatonin and nutritional antioxidants may slow aging processes. The free radical theory of aging postulates that accumulated damage from reactive oxygen species (ROS) over time contributes heavily to the functional decline of cells and tissues. In the brain, for example, increased oxidative damage to neurons and glia is implicated in age-related cognitive decline and neurodegenerative diseases. The circadian system itself is not spared: the SCN and peripheral clock cells suffer oxidative damage with age, which can degrade the precision of the clock’s molecular timekeeping. There is evidence that higher oxidative burden correlates with more circadian rhythm disruption in older organisms, creating a vicious cycle. Encouragingly, experiments show that antioxidant interventions in aged animals (including administering melatonin or vitamin E) can restore some amplitude to circadian rhythms and improve activity/rest pattern stability (Drăgoi et al., 2024a; Hassan et al., 2024; Reiter et al., 2024).

Melatonin is unique among endogenous antioxidants because of its efficiency and ubiquity: it can donate electrons to neutralize radicals, and its metabolites (like AFMK) are also free radical scavengers, yielding a cascade of antioxidant actions. Unlike classic dietary antioxidants (vitamin C, vitamin E) which localize to specific compartments, melatonin being both lipophilic and hydrophilic distributes through all cellular compartments, especially concentrating in mitochondria where the majority of ROS are produced. Mitochondrial DNA lacks many protective histones and is particularly vulnerable to oxidative damage; melatonin’s decline with age has been linked to increased mtDNA damage in aging cells. Supplementing melatonin in aged rodents has been shown to improve mitochondrial respiration efficiency, reduce lipid peroxidation, and even extend lifespan in some studies. For example, older mice given nightly melatonin had levels of oxidative damage markers in brain and liver comparable to much younger mice, and they maintained better learning/memory performance than unsupplemented old mice (Brazăo et al., 2020; Jia et al., 2024; Keskin-Aktan et al., 2022; Zhou et al., 2025).

In the context of healthy aging, melatonin supplementation is primarily discussed as a lifestyle-based strategy aimed at supporting sleep quality and circadian alignment in older adults. In contrast, its use in specific clinical contexts—such as circadian rhythm sleep disorders, neurodegenerative diseases, or metabolic dysregulation—is addressed as an adjunctive therapeutic approach, where evidence remains heterogeneous and often limited to small-scale or short-term studies.

Clinically, melatonin has been explored in neurodegenerative conditions due to its antioxidant and anti-amyloid properties. In patients with mild cognitive impairment (often a precursor to Alzheimer’s disease), prolonged melatonin supplementation reportedly slowed the progression of cognitive decline and improved sleep and mood, though larger trials are needed. In Parkinson’s disease models, melatonin protects dopaminergic neurons from toxin-induced death, likely by reducing oxidative stress and inhibiting apoptotic pathways (Hu et al., 2023; Lv et al., 2024; Sumsuzzman et al., 2021; Zhang et al., 2025). Outside the central nervous system, melatonin’s antioxidant actions may benefit the cardiovascular system, improving endothelial function and blood pressure and immune system, reducing oxidative burst damage during inflammatory responses. Some gerontologists have even proposed melatonin as an “anti-aging” hormone supplement, given the convergence of its effects on multiple aging hallmarks (oxidative damage, chronic inflammation, impaired sleep/circadian regulation) (Barbu et al., 2017; Bondy and Campbell, 2020; Cardinali et al., 2022; Drăgoi et al., 2024a; Favero et al., 2017; Fernández-Martínez et al., 2023; Méndez et al., 2023; Stanciu et al., 2019). A review by Cardinali highlights melatonin’s potential in counteracting neurodegeneration and frailty in the elderly, noting melatonin’s excellent safety profile and low toxicity even at high doses (Cardinali, 2019).

Melatonin supplementation in older adults must be timed correctly to avoid phase shifts that could worsen circadian timing if misused. Generally, melatonin is given 1–2 h before desired bedtime for insomnia or circadian phase advancement; in trials with Alzheimer’s patients, evening melatonin (2 mg prolonged-release) improved sleep but too high a dose or wrong timing sometimes caused daytime sleepiness. Large-scale, long-term clinical trials are still lacking to decisively prove anti-aging benefits or prevention of cognitive decline. Some inconsistent results in past trials (for cognitive outcomes) may relate to using melatonin in advanced disease stages or issues with absorption in older people. Nonetheless, current evidence supports melatonin’s use for improving sleep and circadian alignment in aging, which itself can confer systemic benefits (better sleep can improve memory consolidation, metabolic regulation, and mood, indirectly supporting healthier aging) (Fan et al., 2017; Moslehi et al., 2022; Zhang et al., 2025).

Beyond melatonin, other antioxidants from diet are important in an overall strategy for healthy aging. Vitamins C and E, zinc, selenium, and carotenoids help quench free radicals and are linked to lower incidence of chronic age-related diseases. For example, vitamin E (α-tocopherol) supplementation in the elderly has shown some improvements in immune function and reduction in oxidative biomarkers. Diets high in polyphenols (flavonoids from berries, quercetin from onions, catechins from green tea, etc.) are associated with improved cognitive aging and lower dementia risk, thought to be due to antioxidant and anti-inflammatory effects in the brain. Notably, many polyphenols also exhibit circadian activity – e.g., resveratrol can activate the SIRT1 pathway which influences the clock, and quercetin can modulate clock gene expression in cells. Coenzyme Q10 is another mitochondrial antioxidant that declines with age; supplementation in older adults has shown reduced oxidative stress in tissues and possibly improved endothelial function (Bayu and Wibisono, 2024; Lei et al., 2022; López-Lluch, 2021, 2023; Sun et al., 2015; Xiong et al., 2023).

However, trials of single antioxidant supplements for extending lifespan in humans have largely been disappointing. The consensus now is that a broad-spectrum antioxidant approach via diet (whole foods) is preferable. A Mediterranean-style diet rich in fruits, vegetables, nuts, fish (with omega-3 fatty acids), and olive oil provides a wide array of antioxidant and anti-inflammatory compounds, and has consistently been associated with healthier aging outcomes, including better cognitive function and lower all-cause mortality (Barbouti and Goulas, 2021; Dumitrescu et al., 2025; Scoditti et al., 2022). Such a diet may also beneficially modulate the gut microbiome and circadian metabolism. In fact, a healthy diet and robust circadian rhythms likely reinforce each other: individuals with consistent daily routines and sleep patterns tend to make better dietary choices, and conversely a nutritious diet supports good sleep and energy to maintain activity rhythms (Dong et al., 2020; Finicelli et al., 2022; Zairi et al., 2022).

## 7 Integrating chronobiology and nutrition for healthy aging: future directions

The emerging insights into melatonin, chrononutrition, and antioxidants suggest an exciting integrative approach to healthy aging. Maintaining circadian homeostasis might be a key modulator of aging rate and this can be pursued through lifestyle interventions rather than high-tech medicine. A future vision of geriatric care could include personalized chronotherapy plans: timing of meals, light exposure, exercise, and possibly melatonin administration tailored to each individual’s circadian profile, measured via wearable circadian phase monitors (Cornelissen et al., 2025; Creangă et al., 2025; Ritivoiu et al., 2023, Drăgoi et al., 2024b; Springer et al., 2025). For example, an older person with a blunted melatonin rhythm might follow a schedule of morning outdoor light and evening melatonin or cherry juice, with dinner timed at least 4–5 h before bedtime and composed of sleep-promoting nutrients. These interventions are low-cost and low-risk, and they target fundamental aging mechanisms: sleep/circadian disruption, oxidative stress, inflammaging.

On the research front, several questions remain. While cross-sectional studies show correlations between circadian disruption and age-related diseases, we need more longitudinal studies to determine if circadian improvements can delay the onset of those diseases. Can enhancing melatonin in middle-age, when the decline begins, slow the accumulation of oxidative damage and reduce the incidence of neurodegenerative disease decades later? This is a hypothesis worth testing in long-term trials. Moreover, the optimal dose and formulation of melatonin for older adults require clarification – sustained-release melatonin might better mimic the natural profile and sustain sleep through the night, for instance (Batla et al., 2021; Givler et al., 2023; Thanawala et al., 2024).

Another area of interest is developing or identifying new food-derived chronobiotics. Bioactive peptides from foods like in milk or fermented products might interact with central or peripheral clock receptors. Probiotics could potentially influence circadian rhythms via the gut-brain axis – initial studies show certain probiotic strains can alter neurotransmitters and might improve sleep quality. Conversely, understanding circadian biology might help optimize timing of antioxidant or supplement intake – for example, taking coenzyme Q10 in the morning when mitochondrial activity is higher, or vitamin D at specific times.

Finally, it’s worth noting that aging itself disrupts circadian rhythms, but conversely, experimental circadian disruption (e.g., chronic jet-lag in animal models) can shorten lifespan and induce phenotypes of premature aging (Kumari et al., 2024; Zhang et al., 2025). This bidirectional relationship suggests that keeping the circadian system “young” may keep the body younger. Melatonin appears integral to this process – not only signaling nighttime but also acting as a nightly repair and reset molecule for cells. Melatonin may be a “guardian of the aging clock” helping to preserve the robustness of biological rhythms against the onslaught of time. While melatonin is no magic bullet for aging, in combination with chrononutrition and healthy lifestyle practices, it represents a powerful leverage point to promote healthy aging.

## 8 Limitations and evidence gaps

Key gaps include limited long-term randomized clinical trials, variability in intervention timing and dosing, and insufficient stratification by age, sex, chronotype, and comorbidities. Addressing these limitations will be critical for translating mechanistic circadian insights into evidence-based clinical strategies.

Notwithstanding emerging mechanistic and translational data implicating circadian regulation, melatonin signaling, and chrononutritional strategies

## 9 Conclusion

In this concluding section, the evidence presented throughout the review is integrated into a unified framework linking circadian regulation, melatonin signaling, chrononutrition, and redox homeostasis in the context of aging and healthy aging.

Aging is a complex, multifactorial process, but circadian rhythm disruption, sleep deterioration, oxidative stress, and chronic inflammation are among its central features. Melatonin sits at the nexus of these domains, regulating circadian and sleep physiology while simultaneously providing antioxidant and anti-inflammatory benefits. The age-related decline in melatonin and dampening of circadian rhythms likely contributes to sleep problems, metabolic and cognitive decline, and increased vulnerability to age-related diseases. Conversely, interventions that restore robust circadian oscillations through melatonin, timed light exposure, or meal scheduling, show promise in improving health outcomes in older adults. Chrononutrition offers a practical framework to implement these interventions: by eating in harmony with our internal clock and choosing foods that support circadian and antioxidant functions, we may extend not just lifespan but health span, years of healthy, independent living. Taken together, the evidence reviewed herein supports the view that melatonin constitutes a unifying biological axis through which circadian rhythms, sleep, nutrition, and oxidative balance intersect during aging.

While a substantial body of mechanistic evidence supports the role of circadian regulation, melatonin signaling, and redox balance in aging-related processes, translation to clinical outcomes remains an evolving field. Current human data are largely observational or derived from small-scale interventions, underscoring the need for cautious interpretation and further clinical validation. Moving forward, more research is needed to translate these insights into guidelines. However, current evidence is sufficient to encourage people of all ages to adopt circadian-friendly habits: keep a regular daily schedule, prioritize a dark sleep environment and perhaps a melatonin supplement if needed, get morning sunshine, avoid caffeine and heavy meals late in the day, and enjoy a nutrient-rich diet replete with fruits, vegetables, and other antioxidant sources.

Throughout the manuscript, rejuvenation-oriented terminology has been avoided in favor of functional descriptors that more accurately reflect the current evidence base, including circadian robustness, rhythm amplitude, sleep consolidation, and metabolic alignment.

Importantly, the reviewed evidence supports the view that aging-related circadian changes exist along a continuum from adaptive physiological remodeling to maladaptive disruption. Recognizing this distinction is essential to avoid over-pathologizing aging itself and to appropriately contextualize chronobiological and nutritional interventions as strategies for supporting functional resilience rather than reversing aging.

These interventions should be interpreted within their appropriate translational context, as their relevance differs across populations and clinical settings, emphasizing the importance of personalized application and additional clinical validation.

In summary, melatonin and chrononutrition represent an exciting frontier in gerontology. They exemplify how an understanding of basic circadian biology can be harnessed into accessible nutritional or behavioral strategies that empower individuals to age more healthfully. By synchronizing our lifestyle with our internal biological clock and ensuring that clock is sustained by melatonin and antioxidants, we align ourselves with the rhythms of nature. Such alignment may be one of the keys to unlocking vitality in our later years, helping us not only live longer, but live better.

The evidence reviewed does not support a strictly linear model linking melatonin decline to circadian dysfunction and pathology. Instead, aging-related circadian changes arise from multicausal interactions and feedback mechanisms, with outcomes determined by the balance between adaptive resilience and maladaptive disruption.

Overall, both the timing and dosage of circadian-modulating interventions are critical determinants of efficacy, with timing governing circadian alignment and dosage shaping the magnitude of metabolic and antioxidant responses.

Reinforce consistency by summarizing findings using the aligned terminology (aging, healthy aging, circadian disruption), strengthening conceptual closure.
